# Cocreation of Integrated Interventions Addressing Noncommunicable Diseases and Environmental Degradation: Protocol for a Participatory Qualitative Study

**DOI:** 10.2196/80368

**Published:** 2025-11-25

**Authors:** Nushrat Khan, Paraskevi Seferidi, Kristine Belesova, Nantu Chakma, Laura Downey, Noshin Farzana, Sarada Satyamoorthy Garg, Suparna Ghosh-Jerath, Renu John, Maroof Khan, PK Latha, Asri Maharani, Sabhya Pritwani, Sekar Aqila Salsabilla, Haryani Saptaningtyas, Vidisha Sharma, Sujarwoto Sujarwoto, Aliya Naheed, Vidhya Venugopal, Christopher Millett, Vivekanand Jha, Devarsetty Praveen

**Affiliations:** 1 Primary Care and Public Health Faculty of Medicine Imperial College London London United Kingdom; 2 Non Communicable Diseases Nutrition Research Division International Centre for Diarrhoeal Disease Research Dhaka Bangladesh; 3 University of New South Wales The George Institute for Global Health Sydney Australia; 4 Department of Environmental Health Engineering Faculty of Public Health Sri Ramachandra Institute of Higher Education and Research Chennai India; 5 George Institute for Global Health New Delhi India; 6 School of Health Sciences Division of Nursing, Midwifery and Social Work University of Manchester Manchester United Kingdom; 7 Department of Public Administration University of Brawijaya Malang Indonesia; 8 Percik Institute Surakarta Indonesia; 9 Sebelas Maret University Surakarta Indonesia; 10 Manipal Academy of Higher Education Manipal, Karnataka India

**Keywords:** multisectoral intervention, planetary health, cocreation, noncommunicable diseases, implementation research

## Abstract

**Background:**

Addressing the adverse impacts of climate change on human health requires a global effort across multiple sectors. People living in low- and middle-income countries are particularly vulnerable to the health crises induced by climate change. Therefore, context specific solutions to tackle such challenges are essential to ensure preventive measures are in place for mitigating such risks.

**Objective:**

This protocol aims to outline an integrated, participatory approach to cocreate multisectoral interventions tailored to specific environmental and health challenges in Bangladesh, India, and Indonesia. This work is done as part of the Global Health Research Centre for Non-Communicable Diseases and Environmental Change, funded by the National Institute for Health and Care Research. The overall aim is to collaboratively design and assess interventions that deliver dual benefits for planetary and human health.

**Methods:**

To address the multisectoral nature of the challenges, this study will adopt a cocreation methodology that blends co-design and coproduction approaches. While the problem areas are specific to each context—tackling air pollution due to plastic burning in Indonesia, improving dietary diversity of public food procurement systems and managing extreme heat in India, and mitigating drinking water salinity in Bangladesh—the underlying cocreation framework is consistent and can be adapted to the needs of each study setting. The workflow consists of 4 key stages guided by an existing cocreation framework: planning, developing, evaluation, and reporting, with the 6 core elements of the Medical Research Council’s complex intervention development framework embedded throughout the process. Drawing on the Double Diamond design process, the cocreation stage involves the following phases: codevelopment of a theory of change to explore potential context-specific interventions, short-listing of intervention components through gap analysis and prioritization, co-designing and coproducing selected intervention components, and assessing appropriateness and feasibility of intervention implementation. The cocreation process will be evaluated using the Research Quality Plus for Co‑Production framework to ensure methodological rigor and quality.

**Results:**

Cocreation will take place over 6 months. Sampling and recruitment of cocreators (key stakeholders across sectors) have been completed in all 3 countries, with each cocreator group consisting of 20-30 members. We have developed the tools for the cocreation phase, informed by the findings from formative research, and received the necessary ethics approval to conduct these activities. We will generate a series of academic and nonacademic outputs on the cocreation process to disseminate the findings, as well as training materials for implementers to facilitate future adoption in similar settings.

**Conclusions:**

The cocreation of multisectoral interventions to tackle both environmental change and health is a comparatively new domain of implementation research. This protocol addresses the complex, multidimensional, and unique nature of such interventions by developing a structured and scientifically sound approach to be implemented in real-life settings.

**International Registered Report Identifier (IRRID):**

DERR1-10.2196/80368

## Introduction

Environmental change and its impacts on human lives and livelihoods are a major global concern [1]. The adverse effects of rising global temperatures and extreme weather events are evident in increasing physical and economic losses as well as a surge in noncommunicable diseases (NCDs) [2,3]. Those living in low- and middle-income countries (LMICs) are often among the most vulnerable and disproportionately affected due to their high exposure and sensitivity to the impacts on ecosystems, human habitat, and infrastructure [4,5]. Health systems in many of these countries also have the lowest climate readiness scores, meaning that they are poorly prepared to adapt their available resources to climate vulnerabilities [6]. Given the complexity and scale of such climate change–induced health issues, there is a critical need to develop and implement context-specific solutions through collaboration across different sectors and disciplines, also known as multisectoral interventions [7]. Suited to local needs, these multisectoral interventions can help with early mitigation of risks and reduce the extent of human activities that lead to adverse environmental and health outcomes.

The National Institute for Health and Care Research (NIHR) Global Health Research Centre (GHRC) for NCDs and Environmental Change is a collaborative effort among researchers from 4 countries that aims to achieve dual benefits for health and the environment by developing such multisectoral interventions [8]. NIHR GHRC focuses on a context-specific environmental issue in each geographic region that is important both locally and globally. The first problem area is tackling air pollution from plastic burning in Indonesia, where increasing plastic pollution is a major threat because of both the production and import of large quantities of plastics [9]. The second problem area is managing extreme weather events such as heat waves and improving dietary diversity within public food procurement systems in rural India. Heat waves have been associated with increased risks of dehydration, heat stroke, cardiovascular complications, and mortality, especially among vulnerable populations such as older adults and those with preexisting NCDs [10]. Climate change can negatively impact agriculture and dietary diversity of rural and indigenous populations in such settings, which can worsen malnutrition in all its forms and diet-related NCDs [1,11]. The third problem area is mitigating drinking water salinity in coastal areas in Bangladesh, where surface water and groundwater bodies are becoming increasingly salinized because of rising sea levels and tropical storm surges. Regular intake of such saline water can result in a range of health issues, including hypertension and cardiovascular disease [12,13].

Multisectoral interventions designed to address these interconnected challenges must be grounded in local needs and priorities to be effective across diverse contexts. Guided by the Medical Research Council (MRC) framework [14] for developing and evaluating complex interventions, NIHR GHRC adopted cocreation as a collaborative participatory approach for developing and implementing complex multisectoral solutions [15]. Cocreation is an overarching term that includes both coproduction and co-design [16]. This involves engaging diverse stakeholders across multiple disciplines throughout the process of understanding the issue at hand as well as designing and evaluating contextually relevant solutions [15,16]. While a range of frameworks have been developed to assist with co-design [17-20], including policy co-design [21,22], coproduction [23], and cocreation of interventions [24], there is a lack of evidence that demonstrates how these could be integrated in the MRC guidance to develop a complex multisectoral intervention. Furthermore, given the dual focus on the environment and health in this study, it is important to incorporate planetary health perspectives in the design and evaluation of interventions in a structured way [25,26], following recently published guidance [27]. In addition, engagement and involvement with a range of stakeholders across multiple sectors and settings requires a culturally sensitive and setting-specific cocreation process. Therefore, this protocol outlines a practical, context-sensitive framework for cocreating solutions with the communities and partners involved in NIHR GHRC to guide the development of dual-benefit multisectoral interventions that address NCDs and environmental change.

## Methods

### Study Setting

This study is being implemented in designated intervention and control areas within each participating country. Cocreation activities are limited to the intervention areas, where stakeholders are actively engaged in developing multisectoral interventions. Table 1 presents an overview of the country-specific projects.

**Table 1:** Study setting and problem areas of the National Institute for Health and Care Research Global Health Research Center for Non-Communicable Diseases (NCDs) and Environmental Change.

Country	Study locations	Problem	NCD risk	Potential solutions
Indonesia	Malang district	Air pollution due to plastic burning	Cardiovascular and chronic obstructive pulmonary diseases	Improved waste management to reduce plastic burning
India	Sarguja district, Chhattisgarh	Lack of dietary diversity in public food procurement systems (Integrated Child Development Services program [28] and Pradhan Mantri Poshan Shakti Nirman [29])	Nutrition and dietary health issues	Adaptation of school food menus and procurement processes to enhance dietary diversity
India	Srikakulam district, Andhra Pradesh	Extreme weather events due to heat	Cardiometabolic diseases	Strengthening health systems and community preparedness for heat-related health impacts
Bangladesh	Khulna district	Increased drinking water salinity	Chronic kidney diseases and cardiovascular diseases	Providing access to safe, nonsaline drinking water

### Study Design

#### Overview

We will use a participatory qualitative study design to cocreate the interventions. Throughout the intervention cocreation process, we will follow the MRC framework for developing and evaluating complex interventions. The intervention identification, development, and feasibility assessment will be guided by the six core elements of the MRC framework: (1) consider context; (2) develop, refine, and retest program theory; (3) engage stakeholders; (4) identify key uncertainties; (5) refine intervention; and (6) economic considerations [14].

Stakeholder recruitment for the cocreator groups will be conducted through a nonrandomized purposive sampling to ensure diverse representation across characteristics of interest such as gender, role, socioeconomic status, and stakeholder influence, which are commonly adopted by cocreation studies [24]. The cocreator groups will be involved throughout all stages of the iterative process outlined subsequently. At the end of this process, the key outputs will include a set of contextually appropriate intervention components and corresponding implementation strategies to support effective and efficient delivery.

**Figure 1:**
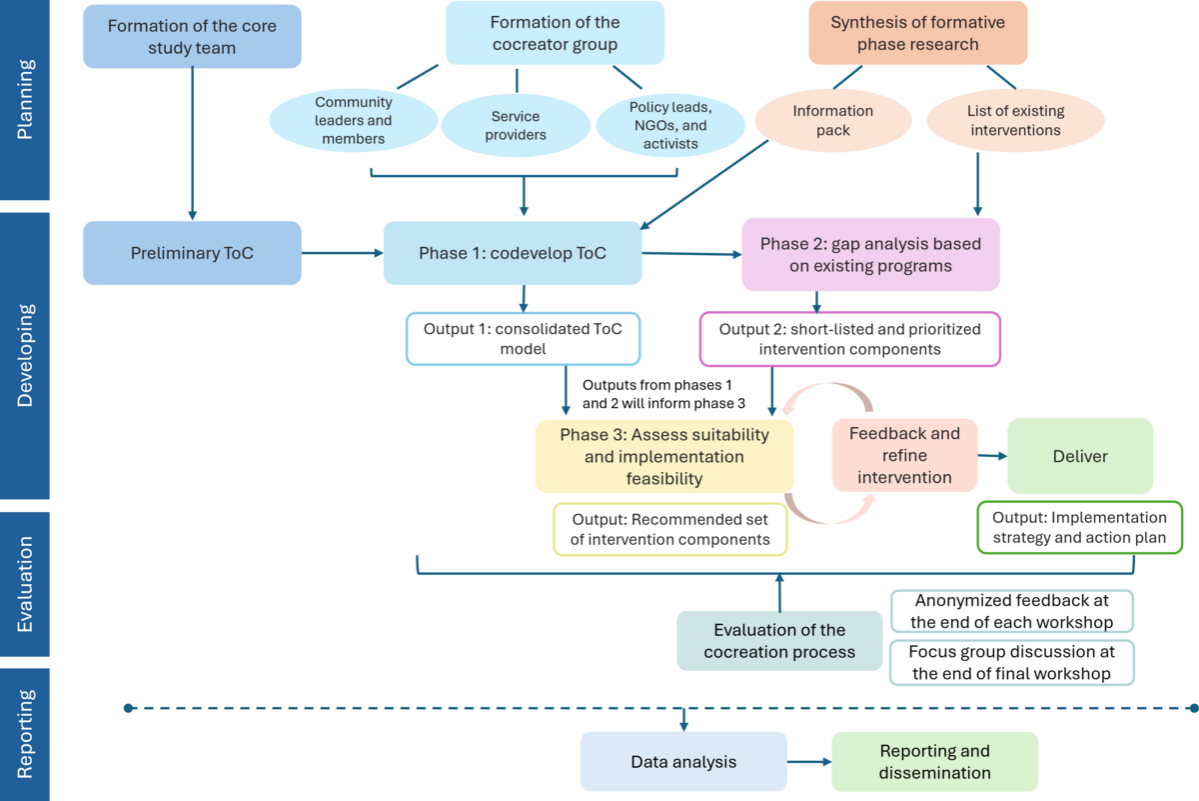
The iterative cocreation workflow for multisectoral intervention development. NGO: nongovernmental organization; ToC: theory of change.

In addition, the cocreation workflow is informed by the framework and principles developed by Leask et al [24], which emphasize participatory engagement across 4 key stages: planning, developing, evaluation, and reporting. Figure 1 illustrates the iterative cocreation workflow adapted for this study.

Table 2 presents the frameworks used in guiding different phases of the cocreation process. While many implementation frameworks exist, these frameworks have been chosen based on their use in existing literature and appropriateness for our desired intervention output.

**Table 2:** Frameworks used throughout different cocreation phases and their purposes.

Cocreation phase	Purpose	Framework used
Overall cocreation process	Guide the overall cocreation process	Medical Research Council intervention development framework [14] and cocreation workflow by Leask et al [24]
Planning	Contextual analysis	Context and implementation of complex interventions framework [30]
Development of the intervention	Guide the intervention design process Pre-evaluate the cocreated intervention through a planetary health lens before implementation	Double Diamond design model [31] Planetary health framework [26]
Evaluation	Evaluate the cocreation process	Research Quality Plus for Co-Production framework [32]
Reporting	Report cocreation outcomes using a structured checklist	Leask et al [24]

#### Planning

The planning stage incorporates preparatory activities to inform the intervention development. The key objectives of this stage are to (1) develop a comprehensive understanding of the context based on the findings from previous formative research, (2) establish a core study team composed of researchers and community engagement members, and (3) recruit cocreators from relevant stakeholder communities to form a cocreation group. The core study team will define the cocreation process, provide necessary training and guidance, and support the recruitment of cocreator group members. The cocreator group will provide active input in the intervention development process and help with the development of implementation strategies.

As emphasized by the MRC framework, the success and outcome of an intervention are strongly influenced by the context in which it is implemented. Contextual understanding can enable, modify, constrain, or facilitate the intervention design and delivery [14,30]. Therefore, an in-depth understanding of the context is crucial for developing an intervention suited to that specific setting. To inform this context, we conducted formative research, including literature and policy reviews, as well as qualitative and quantitative research in each setting. These findings will be synthesized to describe the existing landscape of the issue, local community practices, availability of resources, and stakeholders’ perceptions. We will use the context and implementation of the complex interventions framework to map these findings against relevant contextual domains: geographical, epidemiological, sociocultural, socioeconomic, ethical, legal, and political [30]. We will also consider other independent interventions implemented in the same setting and targeting the same population group, as these may influence the outcomes of the proposed intervention.

To ensure meaningful engagement with relevant stakeholders, core study teams composed of researchers and community engagement members will be formed in each country. These groups will support the preparation of information packages based on synthesized contextual information and training materials, aid in the recruitment of cocreator group members, and advise on specific stakeholder engagement methods.

Guided by these core study teams, stakeholders from the cocreator groups will be purposefully selected, with members across different groups equally represented, including those from vulnerable groups. This will be guided by the stakeholder mapping conducted as part of formative research and community engagement activities. The core study teams will collaborate with the cocreator group members to coproduce rules of participation and facilitate necessary training and knowledge-sharing opportunities.

#### Developing

##### Overview

The development stage includes a series of iterative phases, guided by the Double Diamond design process [31]. The key objectives of this stage are (1) codevelopment of a theory of change to identify potential interventions [33,34] (discover), (2) prioritization and short-listing of existing solutions (define), (3) assessment of suitability and implementation feasibility of proposed solutions through a planetary health lens (develop), and (4) development of implementation strategies to deliver the final intervention (deliver). A series of iterative workshops and interviews will be conducted with the cocreator groups to achieve these objectives. There may be several iterations of intervention refinement and implementation strategies based on stakeholder feedback before piloting and delivering the intervention.

##### Codevelop a Theory of Change Model

A theory of change describes why and how an intervention or program works. Depicted as a causal diagram, the theory of change can be an effective tool to design and evaluate interventions by outlining pathways to achieve intended outcomes, underlying assumptions, and indicators to measure change [34]. We will codevelop our theory of change in 2 stages by involving the core study teams and cocreator groups to ensure it is context specific and culturally responsive [35]. As the first step, the core study team will participate in an exploratory workshop to discuss the problem we are addressing and the scale and ambition of the solutions to aim for. On this basis, they will develop a preliminary theory of change, setting the overarching aim of intervention, identifying its intermediate goals, and outlining potential activities (intervention components) linked to these goals.

In the second workshop, the cocreator group will review and modify the proposed long- and short-term goals outlined in the theory of change to reflect community realities and needs. Similar to the first round, they will then identify a list of possible activities that align with these goals, taking into consideration context, scale, and feasibility. Any additional outcomes proposed will be integrated into the preliminary theory of change model to develop an updated theory of change. This will remain a living document and may be revised as necessary throughout the course of cocreation.

##### Short-Listing of Potential Interventions

Following the development of the theory of change, all identified interventions (activities), both those codeveloped and those identified through literature and evidence mapping, will be compiled. A gap analysis and prioritization exercise will be conducted with cocreator groups in smaller breakout sessions. These sessions will assess the strengths, limitations, and relevance of each option. The key discussion points will then be shared with the whole group to coproduce a short list of interventions, using a relevant prioritization technique. For example, these can include the impact-effort matrix, which prioritizes solutions based on their relative value to end users versus the resources needed to deliver the solution [36], or the must have, should have, could have, will not have (MoSCoW) method, which emphasizes end user requirements to classify solutions into respective groups [36,37].

##### Assessing the Suitability and Feasibility of Short-Listed Interventions

Short-listed interventions will be packaged based on alignment with the theory of change and assessed for suitability and feasibility in 2 steps—first, with community members and health care professionals and second, with policy-level stakeholders and planetary health experts.

In the first step, a workshop will be held to explore the cultural acceptability, perceived effectiveness, and implementation feasibility of each intervention. A decision matrix will be used to guide discussions around each of the following criteria [38]: social validity (cultural relevance, satisfaction, and equity), intervention coherence, and dual health-environmental impact [39,40]. The cocreator group will judge whether each intervention meets the outlined criteria or requires modification. Ideas for alternative solutions will also be sought in case the intervention does not meet the criteria. At the end of this workshop, the working group will incorporate the feedback and refine the proposed intervention package.

In the second step, we will evaluate the co-designed multisectoral intervention through a planetary health lens, informed by the planetary health framework for preimplementation assessment, which we tailored for the purposes of our codevelopment process based on previous theories [25,26] and guidance for assessing acceptability and feasibility [39-41]. This will involve in-depth discussions around health and environmental cobenefits and feasibility of intervention implementation, considering practicality, perceived fit with the infrastructure, cost to organizations and policy bodies, as well as aspects of governance. The final intervention package will be refined based on these assessments.

##### Development of an Implementation Plan

The final step in this stage is to develop a detailed implementation plan. Building on the theory of change, an implementation theory will be articulated, outlining how the desired changes should take effect in the setting for the implementation effort to be successful and how the implementation process will be operationalized. We will map the implementation strategies, that is, a set of methods and activities designed for the specific context, as well as identify the implementation agents, that is, the individuals and organizations engaged in the process [42].

#### Evaluation

The cocreation process will be evaluated continuously to ensure the outcomes generated through the process are representative of cocreators’ opinions as well as suitable, tailored, and valid for end users [23,40]. Short anonymous feedback will be used after each workshop to incorporate participant needs and views while not overburdening them. A structured questionnaire for collecting participant feedback at the end of the final cocreation workshop will be developed using the Research Quality Plus for Co-Production framework [32]. The format will include focus group discussions, individual interviews, and surveys. These tools will be adapted to the needs of each setting, depending on the context, nature of the intervention, and how different groups of stakeholders will be involved in different stages of the cocreation process.

#### Reporting

The outputs from the cocreation process will be reported using a structured checklist such as the one proposed by Leask et al [24], to ensure transparency and consistency. Outputs will include artifacts directly derived from the workshops, such as the theory of change model and prioritized list of interventions, as well as qualitative data from the discussions during cocreation workshops and evaluation. Other outputs include a logic model and implementation strategies that will be developed for the final multisectoral intervention based on the cocreation workshop outputs.

Qualitative data collected during the workshops and evaluation process will be analyzed using hybrid thematic analysis [43] in NVivo (Lumivero) software. Inductive analysis will be used for coding the exploratory discussions from the theory of change building workshop, whereas deductive analysis will be used for the later stages, as those workshops should follow a more structured discussion supported by specific tools and frameworks. Any emerging themes will be captured using iterative inductive coding [44]. All findings will be triangulated with observations and field notes recorded by team members during cocreation activities. These combined data sources will inform the final reporting of the cocreation process and outcomes, providing a rich, contextualized account of the intervention development journey.

### Ethical Considerations

Ethics approvals have been obtained from the research and ethics committee at Imperial College London and ethics committees of the George Institute of Global Health, India; the International Centre for Diarrhoeal Disease Research, Bangladesh; and Brawijaya University, Indonesia (Imperial College Research Ethics Committee reference: 6565249 [India], 6694705 [Indonesia], and 6700946 [Bangladesh]). Informed consent will be collected for all participants before their involvement in the cocreation workshops, and all identifiable data will be removed before dissemination. Where appropriate, participants will be compensated according to local guidelines in the study countries.

## Results

Formative phase research activities started after NIHR GHRC was funded in October 2022. Cocreation workshops as part of this formative research phase will take place over a 6-month period, and the multisectoral interventions are expected to be implemented in each country by the end of December 2025. As part of the planning phase described earlier, core study teams have been established in each country to oversee and coordinate the overall intervention development process. Sampling and recruitment of cocreation group members, informed by research partnerships established with relevant organizations during the formative phase, have been completed in all geographic regions. Each cocreation group consists of 20-30 members depending on the project needs and study location. Due to the iterative nature of the process, findings from each stage may necessitate the involvement of new stakeholders. For example, community members and health care professionals are being recruited first for the cocreation workshops, and the selection of policy and planetary health experts will be based on the initial workshop outcomes. Community engagement activities have helped build trust with the community members and foster a deeper understanding of the local context. On the basis of emerging findings from formative phase research, we prepared tools for each stage of the cocreation process and obtained ethics approvals to conduct these activities.

We plan to produce a series of outputs on the cocreation process to ensure knowledge mobilization. First, we will publish scientific papers for academic audiences as well as present our findings at relevant conferences. The publications will describe the outputs of the cocreation process, outcomes of cocreation evaluation, and how planetary health perspectives were incorporated in the intervention design. Other outputs such as blog posts and videos will be developed for public and policy-level stakeholders and disseminated via NIHR GHRC’s website, social media channels, and other relevant platforms. These outputs are intended to have a broader societal impact by reaching nonacademic audiences. Second, as part of our implementation plan, we will develop training materials for implementers of the codeveloped interventions to facilitate future adoption of these cocreation methods in similar settings. Finally, a formative evaluation report outlining key insights into the cocreation process will be produced for NIHR. This will include an assessment of how effectively the cocreation process supported the development of multisectoral interventions that are reflective of locally articulated priorities.

## Discussion

### Cocreation of Planetary Health Interventions

This protocol outlines the cocreation process for developing complex multisectoral interventions for addressing NCD burden due to environmental change in Bangladesh, India, and Indonesia. While participatory approaches are being increasingly adopted for developing and adapting a range of public health interventions [45,46], very few of these studies have been conducted in LMICs [47] or have incorporated a planetary health lens. The cocreation process described here builds on existing theories and frameworks and brings together a robust set of tools that can be adapted to a range of settings for developing complex multisectoral interventions with dual health and environmental objectives.

While the MRC framework provides general guidance for developing complex interventions and highlights the importance of theory-driven approaches [14], there is limited evidence and guidance on how to translate and transfer such approaches for cocreating complex interventions in low-resource settings [21,34]. Our protocol describes how such interventions can be cocreated in collaboration with the community and other relevant stakeholders, grounded in locally defined priorities and context-specific needs. In addition, most examples of complex intervention development processes tend to have a narrow focus, such as specific health care settings [48], focusing on a certain health care service or model [20,49] or aimed at developing tailored systems or programs [17,50-53]. This protocol provides an overview of developing multisectoral interventions that target both environmental and health domains and thus necessitate the integration of multiple components and sectors.

To accommodate this complexity, the cocreation workflow uniquely combines a range of tools and frameworks. We integrated the Double Diamond model into an adapted cocreation framework, guided by the core components of the MRC framework. By codeveloping a theory of change that helps identify shared solutions together and applying a structured prioritization process, the cocreation approach empowers communities to voice and address their needs and priorities. A key strength of this process is the systematic integration of environmental and health cobenefits while maintaining a balance between top-down and bottom-up approaches in assessing appropriateness and feasibility of implementation. By aligning the interventions with community needs and incorporating feedback from policy and system-level stakeholders, we seek to enhance both feasibility and system integration.

### Limitations

While the cocreation process described in this study is adaptable to different contexts, challenges and limitations remain. The structure of participatory activities and modes of collaboration among stakeholders may depend on local dynamics and culture as well as the climate change issue at hand. For example, frequent flooding in study areas in Bangladesh may limit accessibility and frequency of cocreation activities during the rainy season; therefore, the plans will need to accommodate such factors beyond our control. In case of weak buy-in from local partners, adaptations will need to be made to the intervention development and delivery plans. Geographic distance of intervention areas and travel time may also affect participation, necessitating careful selection of workshop locations to ensure equitable access. Therefore, the suggested process may need to be further adapted to facilitate the maximum level of participation and uphold equity and power balance among all stakeholders throughout the process. The cocreation process described in this study does not include details of the co-design process of any educational and training materials. Given the diverse nature of these multisectoral interventions, we will develop targeted guidance for each setting, depending on available resources and identified needs for additional content.

### Conclusions

This cocreation protocol describes a structured process to guide the development of multisectoral interventions that incorporate dual benefits to health and the environment in LMICs. Given the unavoidable effect of climate change on human health, it is increasingly important to consider planetary health aspects when codeveloping new or adapting existing multisectoral interventions that are integrated within the health system. By cocreating context-specific, community-driven solutions, we recognize and build upon the strengths of local actors, working collaboratively to support their leadership in sustaining these efforts. The cocreation process developed as part of NIHR GHRC can act as a blueprint for developing complex multisectoral interventions and be adapted to diverse settings beyond the work of NIHR GHRC.

## Appendix

Multimedia Appendix 1 Peer review report from the NIHR Global Health Research Centre on NCDs and Environmental Change, National Institute for Health Research (NIHR), UK.

## Abbreviations

GHRC: Global Health Research Centre
LMIC: low- and middle-income country
MoSCoW: must have, should have, could have, will not have
MRC: Medical Research Council
NCD: noncommunicable disease
NIHR: National Institute for Health and Care Research

## Data Availability

Data sharing is not applicable to this paper as no datasets were generated or analyzed during this study.
