# Supplementary material for: Cocreation of Integrated Interventions Addressing Noncommunicable Diseases and Environmental Degradation: Protocol for a Participatory Qualitative Study
Source: JMIR Res Protoc. 2025 Nov 25;14:e80368. doi: 10.2196/80368 (PMC12690285; doi:10.2196/80368)
Supplement: Multimedia Appendix 1 [file resprot_v14i1e80368_app1.pdf]

NIHR | National Institute for Health Research

Professor Vivekanand Jha
Professor Christopher Millett

By Email

CENTRAL COMMISSIONING FACILITY
Grange House
15 Church Street
Twickenham
TW1 3NL

Tel: 020 8843 8000
Fax: 020 8843 8001
Email: ccf@nihr.ac.uk
www.nihr.ac.uk/ccf

29 July 2021

Dear Professor Jha and Professor Millett,

NIHR Global Health Research Centres: Research and Institutional Capacity Strengthening In NCDs: NIHR203247 – NIHR Global Health Research Centre on NCDs and Environmental Change

Further to my previous correspondence, notifying you that your Stage 1 application to the Global Health Research Centres Call had been shortlisted to proceed to Stage 2, please find feedback below from the Funding Committee.

You are advised to address these feedback points in Stage 2 of the application process. Please note that this feedback will automatically be incorporated into the final pdf of the completed Stage 2 application form.

The Funding Committee made the following comments:

- The Funding Committee considered that the application was well described and had a very comprehensive research plan.
- The application was considered to be significant and relevant.
- It was noted that the proposal had strong research capacity strengthening plans. In particular, plans by the research team to prepare people to transition to senior leadership positions were well received by the Funding Committee.
- The Joint Lead applicants were, from the information provided, considered to be outstanding, with strong track records in managing grants for projects on population health and NCDs in LMIC settings.

- The Funding Committee recommended that the following should be addressed in a Stage 2 application:
- Concerns were raised over the lack of detail about the NCDs that the proposed research will focus on. The Funding Committee noted that although the proposal occasionally mentioned chronic kidney diseases and hypertension, it was unclear which NCDs and multimorbidity clusters would be addressed by this proposal. This should be addressed at stage 2.
- The impact of salinity on the NCDs being addressed was not fully articulated - this should be addressed at stage 2.

The Stage 2 application form is now available to complete on the Research Management System and the deadline for submission is **Wednesday 24 November 2021, at 13:00 (BST/GMT).**

Further information on the Global Health Research Centres Call, including guidance notes and frequently asked questions, can be found on the Global Health Research Centres website. If you have any queries with your application, you can contact the Global Health team on 0208 843 8286 or by emailing ghrcentres@nihr.ac.uk.

NIHR and DHSC reserve the right to take a portfolio approach to funding decisions for the Centres scheme, to ensure that the funded cohort is balanced in terms of research focus and geography. All funded applications must have met a quality threshold.

Thank you for your interest in the Global Health Research Centres scheme and I look forward to receiving your Stage 2 application.

Yours sincerely,

Dr Sylvia Anie
Senior Programme Manager - Global Health
